# Face Mask Use Among Commercial Drivers During the COVID-19 Pandemic in Accra, Ghana

**DOI:** 10.1007/s10900-021-01004-0

**Published:** 2021-06-22

**Authors:** Ernest Agyemang, Samuel Agyei-Mensah, Elvis Kyere-Gyeabour

**Affiliations:** Department of Geography and Resource Development, University of Ghana, Accra, Ghana

**Keywords:** SARS-CoV-2 virus, Covid-19, Face mask, Public transport, Accra, Ghana

## Abstract

The paper contributes to unravelling the perceptions of urban commercial drivers regarding their vulnerability to catching the SARS-CoV-2 virus while at work. It further examines how the perception of vulnerability influences personal use of face masks by drivers, as well as on their insistence on appropriate masking behaviour by other persons on-board public transport. Overcrowding and unsanitary conditions in informal public transport in Africa could facilitate the spread of the corona virus. However, the use of face masks, among other enhanced mitigation measures significantly contain and minimize the spread of the virus. Primary data, obtained through surveys at five major public transport terminals in Accra, was analysed and interpreted using the Health Belief Model as an explanatory framework. Results indicate that most drivers have a high vulnerability perception to Covid-19. It further emerged that older drivers, in particular, consistently wore face masks and insisted on other persons in their commercial vehicles to follow suit. Socio-demographic factors, and the need to ensure one’s personal safety and those of loved ones were critical determinants of face mask use among surveyed drivers. The study thus recommends that public awareness campaigns should strategically focus attention on the younger generation of drivers (i.e. 18–39 years) who perceived themselves to be immune to the SARS-CoV-2 virus. Also, the collaborative efforts of state and non-state actors, like the transport operator unions, must be further strengthened if the gains made so far against Covid-19 is to be sustained.

## Introduction

The prevalence, severity, and spread of the acute respiratory syndrome coronavirus-2 (SARS-CoV-2), also known as Covid-19 across the globe has been very phenomenal. As of the last updates on 22nd April 2021, the total number of Covid-19 confirmed cases was over 144 million with over 3 million deaths recorded across the globe [1]. Air travels, and land transport (i.e. buses and rail) in particular, is believed to have led to the diffusion of the SARS-CoV-2 virus from the epicenter of Wuhan, in the Hubei province, to other parts of China, and the rest of the world [2]. Several years ago, ships and trains were major vectors during the Spanish Flu pandemic of 1918 [3]. Covid-19 mitigation measures have generally promoted improvement in the sanitary conditions of public transport through regular disinfections, maintaining and cleaning vehicles regularly and masking up [4]. The use of face masks, in particular, has been touted as efficacious in mitigating the spread of infections by obstructing the transmission of infected droplets [5, 6]. Thus, [6] (p. 9) recommend that “there is a need to understand how masks can be used throughout the day by … *adults (at work)* [emphasis added]. A recent Canadian study [7] (p. 8) also called for “anthropological and sociological research into understanding the *reasons for wearing a mask*” [emphasis added].

This present paper directly responds to this challenge by focusing extensively on the reasons adult commercial drivers have for using face masks when they step out to work in public. This establishes the scholarly relevance of this present paper. Specifically, the paper aims (1) to gauge commercial drivers’ perception of their personal risk of contracting Covid-19 while at work; and (2) to identify how this perception of risk influenced their personal use of face masks as well as their insistence on the compliance to face mask use by other persons on-board public transport. Identifying the rationale for masking up can assist health authorities to develop targeted interventions to defeat Covid-19.

## Contextual Issues

In Africa, the hardest-hit countries, in the order of severity of cases, are South Africa, Egypt, Tunisia, Morocco, Ethiopia, and Algeria [8]. Covid-19 incidences in Africa appears to be relatively low, with a mere 3.1% and 3.9% respectively the total global confirmed cases and deaths as of 22nd April 2021 [1, 9]. Thus, some have expressed optimism that the devastation from Covid-19 “may never come” [10] (p. 286). However, an epidemiological model designed at the onset of the pandemic predicted some startling socio-economic outcomes for Africa.

**Fig. 1:**
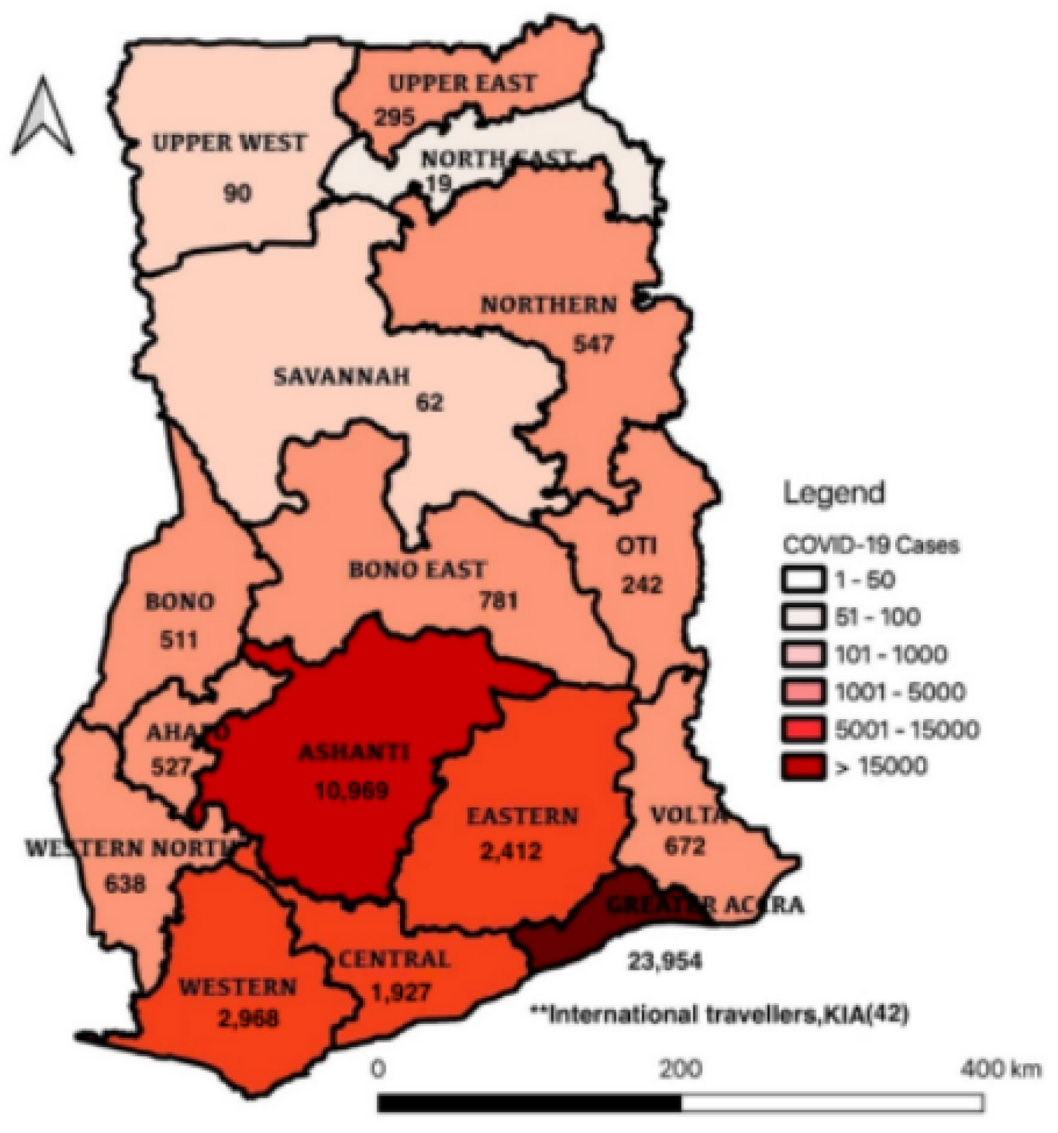
Regional distribution of cumulative Covid-19 cases in Ghana, March-September, 2020. Source: MOH (2020b)

As cited in a [11] report, Africa may witness over 1 million Covid-19 infections, 22.5 million hospitalizations, and 3.3 million deaths, depending on the intervention measures taken to stop the spread. Also, some 19 million job losses are expected, making Africans poorer. Thus, the race is on to contain the community spread of Covid-19 among African countries, including Ghana, which recorded its first two imported cases on 12th March 2020 in the national capital city of Accra [12]. Prior to this, an emergency preparedness and response mechanisms had been planned [13]. Following from this, and as a precautionary measure, the government instituted a partial lockdown of Accra and Kumasi on 29th March 2020. These two cities were identified as critical hotspots for the spread of the virus at the initial phase of the outbreak, and continue to lead current active Covid-19 cases and deaths (see Fig. 1).

The partial lock down was eventually lifted on Monday 20th April 2020 with the announcement of several enhanced Covid-19 mitigation measures by the government. Specifically for public transportation, the measures generally included physical distancing in vehicles, proper handwashing and sanitizing, and use of face masks [14]. In order to comply with the physical distancing directive, drivers of commercial vehicles such as taxis that normally seated 1–3 passengers were now required to allow only two passengers to be seated on a row. Similarly, commercial drivers who hitherto were carrying 1–4 or 1–5 passengers on a row had to reduce the number of passengers to three on a row. In addition, all transport terminals were required to make available adequate running water basins, and sanitizers for the use of passengers before boarding [15]. This follows the donation of sanitary items to various public transport unions in Accra by the Transport Ministry and other corporate institutions [16]. Also, the Transport Minister also urged drivers to take contact details of all passengers to aid future contact tracing, if need be. The Greater Accra Regional Security Council (RESEC) also issued a directive insisting that “notices of ‘No face mask, No entry’ should be visibly displayed at vantage points including lorry stations to mitigate the spread of the SARS-CoV-2 virus [17].

The justification for adopting these mitigation measures is that the insanitary and crammed conditions of public transport could expose Ghanaians to the Covid-19 virus [18, 19].

## Literature Review

Since 2020 when Covid-19 was designated a respiratory pandemic, the universal usage of face masks has gained prominence in most parts of the world. A recent study has shown that about 99% of national governments and health authorities have recommended the use of face masks for all citizens whenever they go to use public places or use public transport [20]. Adequate measures have also been put in place to educate and promote mask use. In some jurisdictions, it is an offence to refuse to wear face masks in public. This is in response to the growing scientific evidence that links face masking and the prevention of spread of respiratory diseases. Several years ago, [21] wrote about that widespread use of face masks as a principal preventive mechanism dating back to the 14th century when the world battled to control the transmission of infected droplets associated with the pneumonic plagues [6] (p. 9) have therefore concluded that “when used in conjunction with widespread testing, contact tracing, quarantining of anyone that may be infected, hand washing, and physical distancing, face masks are a valuable tool to reduce community transmission”.

Meanwhile in Africa, emerging scholarly debates on Covid-19 have generally focused attention on the economic fallouts from the lockdowns due to the nature and scope of informality on the continent [22, 23, 24]. Also, [25] studied on the mobility changes and living arrangement transformations of family and social relations who accommodated returnee kin during the early stages on the pandemic. This study underscores the coping mechanisms and resilience of the African in the face of a global tragedy. Others have also commented on the ‘African COVID-19 anomaly’ which attributes the low African Covid-19 disease and deaths partly to lessons learned from historical experiences with pandemics on the continent [10]. Yet still, others have examined the geopolitical distribution of specialized health care facilities and its role in facilitating or inhibiting prudent Covid-19 emergency response [26]. Specifically, in Ghana, studies have interrogated the efficacy or otherwise of government’s free water intervention to promote proper hygiene and sanitation during COVID-19 [27]. The socio-economic impacts of the COVID-19 pandemic in marketplaces as witnessed in soaring food prices, forceful relocation and decongestion exercises to enforce social distancing among Ghanaian traders have received attention [28]. Two additional Ghanaian studies have examined the ecological readiness and compliance to hand washing, and social and physical distancing recommendations in selected public transportation stations in the Greater Accra Region [29] and in Kumasi [19]. The results of the cross-sectional roadside observations in Kumasi, for instance, indicated that compliance with face masks wearing was as low as 12.6% indicating “the existence of a significant gap in the implementation of the policy on face masks and the need for much more effective implementation of the policy” [19] (p. 6). Going forward, it is important to ask: what is the current state of mask use among Ghanaians and which factors account for this phenomenon? This present paper provides answers to these questions.

## Conceptual Framework

This paper finds inspiration in the Health Belief Model (HBM) which is an explanatory framework developed to account for health-related behaviors of individuals. According to [30] (p. 328), the “Model grew out of a set of independent, applied research problems with which a group of investigators in the Public Health Service were confronted between 1950 and 1960. Thus, the theory and development of the Model grew simultaneously with the solution of practical problems”. The Model posits that an individual's belief in a personal threat of an illness or disease, and in the efficacy of a recommended health behavior or action will predict the likelihood that that individual will adopt a proposed behavior. Health behaviors and response to proposed interventions are as a result of the interplay among factors such as person’s perceived threat to sickness or disease (perceived susceptibility), the belief of consequence (perceived severity), and the potential positive benefits of action (perceived benefits). The rests are perceived barriers to action, exposure to factors that prompt action (cues to action), and confidence in the ability to succeed (self-efficacy). Also, socio-demographic profile of an individual as well as psychological factors, including his or her personality and pressure from his or significant others are known to influence health-related behavior. The HBM has been used extensively, including an exploration of the knowledge, attitudes and preventive practices among Pakistanis in response to a dengue outbreak [31] and the spread of respiratory infections among Saudis [32]. These studies concluded that high perception of vulnerability to diseases resulted in preventive behaviors such as regular hand wash with water and soap, as well as covering of noses and mouths with tissue paper when sneezing or coughing.

Thus, in order to fully understand the key determinants of mask use among commercial drivers in Accra, we explore the interplay among these demographic variables and psychological factors and how they can influence the decision or action taken by individuals in relation to their health.

## Methods

Before the conduct of primary data collection, the study protocol was reviewed and approved by the Ethics Committee for the Humanities at the University of Ghana (ECH-031/20–21). A vox pop survey was also conducted on September 2nd 2020 among selected commercial drivers and the feedback was used in revising the original questions to elicit appropriate responses from participants of the actual surveys. Primary data was obtained through a face-to-face administered survey between 7 and 11th September 2020. The survey tool measured drivers’ level of compliance or otherwise to the use of face masks, the key determinants and background details of participants such as age, education, marital status, etc., as well as fleet operational characteristics. At the time of conducting the survey, the restrictions on in-vehicle physical distancing had been reversed. Observations were undertaken on the availability of facilities for hand washing and their use by the travelling public, but the results are not captured in this present paper.

**Fig. 2:**
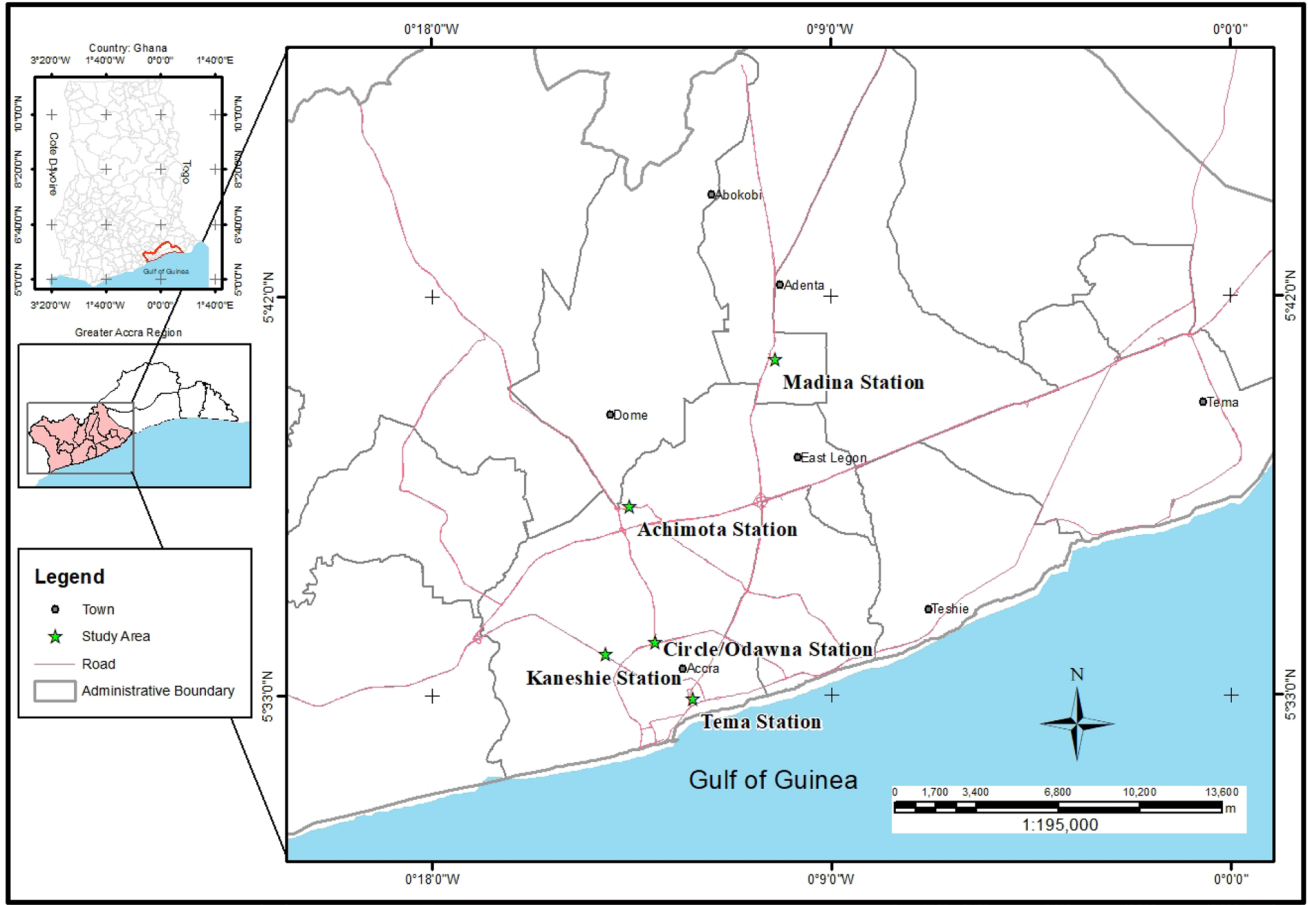
Map of study sites. Source: Author construct, 2020

Research participants were purposively sampled at five public transport terminals in Accra, (5.556° N and 0.1969° W), which is home to an estimated 5 million inhabitants [33]. For many of these residents, informal paratransit services remain popular forms of mobility for work and non-work related trips [18]. Among the busiest and most popular public transport terminals where paratransit services are organized are the Achimota, Kaneshie, Circle/Odawna, Tema Station, and Madina terminals respectively (Fig. 2). The coming together of such large number of passengers at these terminals may be fertile grounds for the spread of infectious diseases like Covid-19, hence their selection as study sites for this paper. Each of the selected study sites was assigned a quota of 100 participants.

While at the respective terminals, the research team randomly selected participants who were drawn from various ‘stations’ or destinations within the terminal until the required quota was met for the day. The focus was on intra-urban commercial drivers, so all inter-urban commercial drivers were excluded from the study. The primary data were analyzed using IBM SPSS statistics version 25. Analysis performed included descriptive statistics, cross-tabulation, mean comparison, and Pearson correlation of various variables that help explain face mask use by commercial drivers.

## Results

Commercial driving in GAMA is generally male dominated, and in this particular study, all respondents were males. Most of these drivers were aged between 25 and 54 years. About 76.2% of drivers had been employed by car owners. On average, most drivers earn about GHS 500 (USD 86) and GHS 1000 (USD 171) per month, out of which their ‘daily sales’ (i.e. fees paid to car owners) and ‘chop money’ (i.e. daily allowance/income for the driving crew) are deducted. The data further shows that most drivers (74.6%) had at least completed their basic education; were married (67.4%) and were responsible for the upkeep of about four to five other persons in the household. Table 1 highlights the key profile of survey participants.

**Table 1:** Respondents’ socio-demographic characteristics

Items	N = 500	Percent
*Age group*		
18–24	16	3.2
25–39	208	41.6
40–54	215	43.0
55 and above	61	12.2
*Vehicle usage type*		
Trotro	440	88
Taxi	60	12
*Vehicle ownership status*		
Driver only	381	76.2
Driver & Owner	119	23.8
*Educational status*		
No formal education	22	4.4
Basic school (Primary—JHS)	373	74.6
Secondary/Technical	93	18.6
University/Polytechnic	12	2.4
*Marital status*		
Single	94	18.8
Married	337	67.4
Co-habiting	44	8.8
Divorced	21	4.2
Widowed	4	0.8
*Household size*		
1–3 members	150	30.0
4–5 members	191	38.2
Above 5 members	159	31.81
*Monthly income (GHC)*		
500–1000	172	34.4
1000–1500	129	25.8
1500–2000	105	21.0
2001 & Above	94	18.8

Even though all category of persons and occupations are vulnerable to the SarsCov2 virus, commercial drivers, their attendants and commuters in general have a higher chance of infection due to overcrowding in public transport, and other activities including handling and exchanging money. It is therefore imperative to identify factors associated with Covid-19 mitigation behavior with a focus on face mask wearing.

In response to the question ‘*did you perceive yourself to be susceptible to Covid-19 around March 2020 when cases in Ghana were rising?*’ as high as 93% of survey participants responded in the affirmative. However, in response to the question ‘*today, as you respond to this survey, do you perceive yourself to be susceptible to Covid-19?*’, there was a 39.6% reduction in the number of ‘yeses’ from the same respondents, indicating that somehow their levels of anxiety to the pandemic had subsided considerably. This notwithstanding, survey participants reported that they still wear their face masks whenever they go out to work as a precautionary measure (Table 2). When the data is split into operator type, Trotro drivers who carry relatively more passengers per trip reported slightly more consistent wearing of face masks compared to taxi drivers who carry fewer passengers.

**Table 2:** Self-reported use of facemasks by commercial drivers (N = 500)

Category	Responses	Percent
All (N = 500)	Always	92.4
	Sometimes	6.8
	Never	0.8
Operator type		
Trotro (n = 440)	Always	92.7
	Sometimes	6.6
	Never	0.7
Taxi (n = 60)	Always	90
	Sometimes	8.3
	Never	1.7

To gauge exactly how much fear of the possible risk of Covid-19 infection related to adherence to the enhanced hygienic protocols, notably facemask wearing, a Pearson product-moment correlation coefficient was performed. The variable “*the risk of drivers like myself contracting Covid-19 whiles doing our business is high”* was tested against the dependent variable “*personally, do you use the face mask whenever you go out to work as a driver?”*. Preliminary analyses were performed to ensure no violation of the assumptions of normality, linearity, and homoscedasticity.

The results indicate a strong, positive correlation between the fear of possible infection with Covid-19 while working as commercial drivers and face mask-wearing, r =  − 0.11, n = 500, p < 0.05. This suggests that when the fear among drivers of getting infected with Covid-19 increases, their tendency to consistently use their face masks also increases, and vice versa. Even though no statistical association was found between age and compliance to face masking, the frequency of older persons (aged above 40 years) reporting consistent use of face masks is relatively high (Table 3). In particular, all the drivers in the 55 years and above age group reportedly wore their face masks whenever they stepped out to work.

**Table 3:** Self-reported face mask use by commercial drivers of different age groups (N = 500)

Age group	Responses	Per cent
18–24; **(***n* = 16)	Always	75.0
	Sometimes	18.8
	Never	6.3
25–39; (*n* = 208)	Always	90.9
	Sometimes	8.7
	Never	0.5
40–54; **(***n* = 215)	Always	93.0
	Sometimes	6.0
	Never	0.9
55 years and above; (*n* = 61)	Always	100.0
	Sometimes	0.0
	Never	0.0

In response to the question, ‘*as a precautionary measure, do you insist on all passengers [and your mate] wearing their face masks at all times in the vehicle?*’ most drivers (91%) responded in the affirmative. This implies that most drivers, especially those who started their trips from the transport terminals, prevailed on passengers and also their assistants/mates to put on their face mask before departure. Perhaps not so surprising, the data suggests that among drivers who were routine users of face mask, a majority of them (93%) also ensured that all persons who boarded their vehicles equally used the face mask. The statistical analysis demonstrates a strong, positive correlation between the personal consistent use of face mask by drivers and the insistence to get commuters and drivers’ mates to wear their face mask, r =  − 0.28, n = 500, p < 0.01. Also, the results from Pearson correlation of enforcement of face mask use and respondents' socio-demographic characteristics indicate that age was statistically significant and negatively correlated to the dependent variable (r = − 0.14, n = 500, p < 0.01). This indicates that older drivers are more inclined to insist on compliance to face mask use by persons who board their vehicles, relative to young drivers.

The study set out to test the null hypothesis that states that sociodemographic and psychological factors such as age, level of education, driver type and years of driving experience, perceived vulnerability and personal safety cannot predict face mask usage as a mitigation measure against Covid-19 spread among commercial drivers in Accra. This was done to assess the extent to which these demographic variables and psychological factors interplay to influence the action individuals take in relation to their health, as espoused by the HBM. Regarding the use of face mask by commercial drivers, the result of the binary logistic regression showed that personal safety (p > 0.05), perceived vulnerability (p > 0.05), years of driving experience (p > 0.05), group pressure from family and friends (p > 0.05), education status (p > 0.05) and age of respondents (p > 0.05) were not significant predictors.

However, driver operator type (p < 0.05) and the insistence on masking up by car owners/transport unions (p < 0.05) were significant predictors of personal use of face masks by surveyed commercial drivers in Accra. For example, the data shows that the likelihood of drivers masking up increases amongst trotro drivers (β = 1.70, p < 0.05) compared to taxi drivers. Trotro drivers are 5 times more likely (OR = 5.45, CI 1.26–26.60) to mask up compared to taxi drivers. Further, in situations where car owners/transport unions do not insist on drivers masking up, drivers were 18 times less likely (OR = 1/0.055) to personally wear their face mask.

With reference to drivers’ insistence on car occupants to mask up, the results of the logistic regression showed that driver type (p > 0.05), perceived personal safety (p > 0.05), perceived vulnerability (p > 0.05), driving experience (p > 0.05), pressure from colleagues at work (p > 0.05), and education (p > 0.05) were not significant predictors. Nonetheless, pressure from family and friends (p < 0.05), as well as employers’ insistence (p < 0.05) and age (p < 0.05) were significant predictors of the probability of drivers to insist that other car occupant mask up. Specifically, drivers within the ages of 25–39 years were 17 times less likely (OR = 1/0.059) to insist on car occupants masking up compared to drivers above 55 years. Further, drivers whose employers do not insist on their masking up are 3 times less likely (OR = 1/0.38) to insist on other car occupants’ masking up. Lastly, drivers who are pressured by their family and friends to mask up are 57 times more likely (OR = 57 times, CI 12.26–265.48) to insist on other car occupants masking up.

In order of importance, Table 4 illustrates the specific reasons adduced by respondents for consistently masking up and also insisting that other car occupants mask up as well. The reason, ‘*I do so for my own safety and those of my loved ones*’ yielded the highest affirmative response (94%) toward face mask use among drivers. The response ‘*I see other members of the public wear their face masks*’ was considered the least determinant for face mask use among surveyed participants. Table 4 summarizes the major reasons respondents have for consistently wearing face masks.

**Table 4:** Reasons for ALWAYS wearing a face mask in per cent (N = 462)

Reasons	Yes	No
I do so for my own safety and those of my loved ones	94	6
The President has urged me to do so	68	32
I can be prosecuted for not wearing a face mask	67	33
The Greater Accra Region has enforced a compulsory ‘No Mask, No Entry’ policy in public places	65	35
My immediate family members insists/reminds me to do so	61	39
My employer/operator union insists/reminds me to do so/provides masks for me	58	42
I see other members of the public wear their face masks	30	70

In spite of the perceived benefits that motivate drivers to mask up, a few of the respondents (n = 34) admitted to never using face masks or using them occasionally. The main barriers to adoption of face mask include statements like ‘*the virus is no longer as frightening now as it used to be initially’ (100%); ‘it is not comfortable wearing face mask’ (70%))* and *I trust God to protect me even without wearing a mask (25%).*

## Discussion

This study provides evidence to support an earlier study that found a relationship between high perceptions of health anxieties and precautionary behavior [34]. In the present study, it emerged that most drivers surveyed felt they were highly susceptible to catching the virus, especially during March 2020 when the disease first surfaced in Ghana. This may be due to the risk factor of occupational exposure as drivers and their driving crew make regular contacts with the travelling public [35]. It has been argued that the anxiety/precautionary behavior relationship is a lot more complex and involves multiple pathways of competing directionality [36]. However, the perception of vulnerability seem to ebb away in the subsequent months when people become accustomed to the so-called ‘new normal’.

Observations made at the transport terminals months after the onset of Covid-19 show that actual usage of face masks was quite minimal. At the time of the survey, most drivers were not seen wearing their face masks. When questioned by the researchers, such drivers were quick to show us their face masks to indicate that they have the masks nearby and explained that they had briefly taken them off to eat, or relax and chat with fellow drivers who were waiting their turn to set off. At our insistence, some put on the masks for the interviews to be conducted, and others simply promised that they will wear them when their vehicles are full and they are existing the terminals. Such relaxation of health behavior is not unique to this study. According to [37], most of Iranian adolescents typically underestimate their risk perception of being infected by the COVID-19 virus. Similarly in China [38], report that drug supply and patients’ compliance in quarantined communities have faced lots of challenges months after the disease outbreak.

This present study could not find statistical variation among surveyed drivers in terms of their compliance to masking up according to their socio-demographic groups. A possible explanation to this may be due to the fact that usually at the initial phases of pandemic, the level of understanding about the very nature and mode of communal spread of the deadly virus is minimal. Thus, irrespective of one’s socio-economic and demographic background, people are generally anxious. For instance, in commenting on the impact on the Spanish Flu on the psyche of American [39] (p. 42), hit the nail right on the head when she argues that “the influenza virus itself was not selective in its victims, belying notions of gender, class, and racial superiority…anyone might become ill”. This could explain why everyone felt the need to heed to the precautions announced.

Having said this, it is important to point out that, in the study, all the drivers in the 55 years and above age group reportedly wore their face masks whenever they stepped out to work. This corroborates an earlier study that concluded that older persons were more anxious about the spread of Covid-19 due to possible worsening of their physical status should they get infected [40].

Again, we concur with a recent article by [41] who avers that media attention in recent years has “shaped outbreak coverage in various ways, heightening alarm while serving as a useful tool for encouraging precautions and prevention”. In the case of Ghana, the rising Covid-19 case counts and associated deaths in the country and beyond was given extensive coverage in both the traditional media outlets, notably radio and television, as well as on social media. Also, regular updates on the Covid-19 situation by the president of Ghana himself accompanied with his incessant pleas for the citizens to mask up was also given priority media attention. Thus, it is reasonable to expect the adoption of precautionary behavior by most Ghanaians, including the surveyed drivers.

Another important factor that explains the high compliance among trotro drivers to face mask-wearing is the critical role played by the transport Owner and Driver Unions, the largest of which is the Ghana Private Road Transport Union (GPRTU) [42]. The Unions are known to strictly control the activities and conduct of drivers at major terminals in Ghana [43]. A special task force of the GPRTU was observed to physically inspect that all vehicle occupants wore their face masks before vehicles are allowed out of the terminals. Passengers who inadvertently forgot to carry their face masks along were compelled to either buy new face masks at the terminals or were refused entry into the vehicles. The enforcement of face mask use at the point of departure may explain the relatively high use of face mask among trotro drivers, compared with taxi drivers who may not belong to the GPRTU.

As a result of reduced anxiety, some participants simply refused to take precautions against getting infected or transmitting the virus. Among drivers who reported that they seldom use face masks, it could stem from the viewpoint that despite their previous risky behaviors, including never wearing face masks or occasionally wearing them, for instance, when they need to outwit the taskforce team, they never got ill during the height of the outbreak of Covid-19 in Accra. Similar to [40], these drivers, therefore, did not see any reason to amend their behavior at a time when, in their view, the virus has been ‘defeated’.

## Limitations

As in all studies, this present study must be read with some limitations in mind. At the time of conducting the survey, the directive on face mask use by all persons on-board public transport seemed to be the only most publicly enforceable Covid-19 mitigation measure. The others, including social distancing on commercial vehicles, for instance, was no longer enforceable. Also, the directive to provide adequate public hygiene facilities for use by passengers at the terminals had largely been de-emphasized, with the interest now shifting to the use of personal hand sanitizers which were readily available on the market at the time. Thus, the study did not focus on these mitigation directives. Despite these limitations, by strategically focusing our study on face mask use, we believe the findings and recommendations are of policy relevance for Ghana, and other areas with similar public transport characteristics in the quest to defeat Covid-19.

## Conclusions

To conclude, the two-fold objectives of the paper was to ascertain commercial drivers’ perception of their personal risk of contracting the SARS-CoV-2 virus while at work; and also, to identify how this perception of risk influenced their personal use of face masks as well as their insistence on the compliance with use of face masks by other persons on-board public transport. The evidence suggests high knowledge of the disease, and increased awareness of the occupational hazard associated with Covid-19 infection among survey participants. The anxiety was such that, most participants, particularly older drivers complied with the face masks directive to mitigate the spread of Covid-19 [5, 6]. Besides, the good behavior of these drivers was transferred to other persons on-board public transport as passengers and drivers’ mates were compelled to put on their face mask prior to departure. Aside socio-demographic factors, personal safety and those of loved ones were critical determinants of face mask use. On the downside, less anxiety about the pandemic in recent times explains the erratic or complete non-use of face masks. Ghana, and for that matter Africa is not out of the woods yet in so far as the pandemic is concerned [14].

The recent resurgence of Covid-19 elsewhere should be a wake-up call for the Africa to guard against seeming complacency. To this end, while cautiously celebrating the successes chalked in the management of the pandemic in Africa [10], due in part to the critical role played by public transport operators and users, as has been found in this study, the study recommends that the coordination between state and non-state actors, in this case, the driver unions be further strengthened to sustain the gains made against the pandemic so far. Also, awareness creation on face mask use through various media outlets should strategically focus attention on the younger generation (i.e. 18–39 years) who perceived themselves to be immune to the SARS-CoV-2 virus. This is because this age cohort can serve as vectors of the virus to the detriment of older persons they may come into contact with. The health campaigns on face mask use should highlight the personal health benefits, and that of the protection of one’s immediate family and friends.
